# *Megalobrama amblycephala* IL-22 attenuates *Aeromonas hydrophila* induced inflammation, apoptosis and tissue injury by regulating the ROS/NLRP3 inflammasome axis

**DOI:** 10.3389/fimmu.2024.1447431

**Published:** 2024-08-15

**Authors:** Zhensheng Wang, Wenya Zhai, Hong Liu

**Affiliations:** ^1^ College of Fisheries, Key Lab of Freshwater Animal Breeding, Ministry of Agriculture and Rural Affair/Key Lab of Agricultural Animal Genetics, Breeding and Reproduction of Ministry of Education, Huazhong Agricultural University, Wuhan, China; ^2^ Engineering Research Center of Green Development for Conventional Aquatic Biological Industry in the Yangtze River Economic Belt, Ministry of Education, Wuhan, China

**Keywords:** interleukin-22, bacterial disease, antioxidant, antiapoptotic, anti-inflammatory

## Abstract

Mammalian interleukin-22 (IL-22) attenuates organismal injury by inhibiting reactive oxygen species (ROS) and impeding the NLRP3 inflammasome activation. However, the role of fish IL-22 in this process remains unclear. We characterized MaIL-22, an IL-22 homolog in blunt snout bream (*Megalobrama amblycephala*). Despite its low sequence identity, it shares conserved structures and close evolutionary relationships with other teleost IL-22s. Furthermore, *Aeromonas hydrophila* (*A. hydrophila*) infection leads to tissue injury in *M. amblycephala* immune organs and concomitantly altered *Mail-22* mRNA expression, suggesting that MaIL-22 was involved in the antimicrobial immune response. To explore MaIL-22’s biological functions, we produced recombinant MaIL-22 (rMaIL-22) protein and demonstrated it significantly enhanced the survival of *M. amblycephala* post-*A. hydrophila* infection. To unravel its protective mechanisms, we explored the ROS/NLRP3 inflammasome axis and its downstream signaling responses. The results showed that rMaIL-22 treatment significantly elevated antioxidant enzyme (T-SOD, CAT and GSH-PX) activities to inhibit MDA activity and scavenge ROS in visceral tissues. Meanwhile, rMaIL-22 impeded the activation of NLRP3 inflammasome by suppressing NLRP3 protein and mRNA expression. This indicated that rMaIL-22 contributed to inhibit *A. hydrophila*-induced activation of the ROS/NLRP3 inflammasome axis. Consistent with these findings, rMaIL-22 treatment attenuated the expression of proinflammatory cytokines (*il-1β*, *tnf-α* and *il-6*) and proapoptotic genes (*caspase-3* and *caspase-8*) while promoting antiapoptotic genes (*bcl-2b* and *mcl-1a*) expression, ultimately mitigating tissue injury in visceral tissues. In conclusion, our research underscores MaIL-22’s key role in microbial immune regulation, offering insights for developing IL-22-targeted therapies and breeding programs.

## Highlights

- MaIL-22 exhibits a distinct sequence but maintains conserved structure in teleost.
- rMaIL-22 enhances resistance to *A. hydrophila* in *M. amblycephala*.
- rMaIL-22 inhibits *A. hydrophila*-triggered ROS/NLEP3 inflammasome axis activation.
- rMaIL-22 exhibits potent antioxidant, antiapoptotic and anti-inflammatory actions.

## 1 Introduction

The escalating demand for high-quality fish protein is propelling the expansion of aquaculture globally (1). From a global perspective, China is one of the highest fish consuming countries. Furthermore, freshwater fish is the main source of quality protein in Chinese diet (2). Blunt snout bream (*Megalobrama amblycephala*) is an important species in China’s freshwater aquaculture, reaching 767,343 tons produced in 2022 (3). However, the intensification of *M. amblycephala* farming and the broader expansion of farming scale have led to a notable rise in bacterial diseases, resulting in significant economic losses (4). Among these pathogens, *Aeromonas hydrophila* (*A. hydrophila*), as a gram-negative bacterium, is the main pathogenic bacteria causing bacterial septicemia in fish, including *M. amblycephala* (5). Nevertheless, conventional drug treatments often entail environmental contamination and residues in farmed organisms (6). Therefore, acquiring superior strains of *A. hydrophila*-resistant *M. amblycephala* is crucial for advancing sustainable fish farming and promoting environmentally friendly aquaculture practices.

The immune system is the main line in defense against bacterial invasion (7). Interleukin-22 (IL-22), a member of the IL-10 cytokine family, emerges as a pivotal regulator of host defense and tissue homeostasis (8). IL-22 is present in all jawed vertebrates and is produced by a variety of immune cells, such as Th17 and Th22 cells (9). IL-22 homologs have been identified in several fish, such as zebrafish (*Danio rerio*) (10), puffer fish (*Fugu rubripes*) (11), mandarin fish (*Siniperca chuatsi*) (12), and rainbow trout (*Oncorhynchus mykiss*) (13). Mammalian and fish studies collectively show that IL-22 is an important regulator of the mucosal barrier and contributes to mucosal tissue repair (14–17). Differently, IL-22 may play a pathogenic role in the development and progression of inflammation in mammals. An earlier study indicated that IL-22 exacerbates Zika virus-induced encephalitis by hindering the immune system (18). Furthermore, IL-22 inhibits schwann cell proliferation and exacerbates nerve injury (19). These results suggest that IL-22 plays a diverse role in immune regulation. However, the comprehensive role of IL-22 in fish resistance to *A. hydrophila* infection remains largely unexplored, especially in tissues other than mucosal tissues.

Previous studies have demonstrated that *A. hydrophila* induces oxidative injury, inflammatory responses and apoptosis by elevating reactive oxygen species (ROS) levels in tissues, ultimately leading to tissue injury (20, 21). NLRP3 inflammasome is a high-molecular-weight protein complex, included sensor NLRP3, the adaptor PYD and CARD domain-containing (PYCARD/ASC) and cysteinyl aspartate specific proteinase-1 (Caepase-1), can be activated by ROS and lead to overproduction of pro-inflammatory factors (such as IL-1β and TNF-α), thereby exacerbating the inflammatory response and apoptosis (22, 23). Consequently, the ROS/NLRP3 inflammasome axis may be an important pathway for organismal injury induced by *A. hydrophila*. It is noteworthy that, under conditions of bacterial infection, the antioxidant capacity of IL-22 is believed to play a vital role in maintaining the homeostasis of the internal environment in mammals (24, 25). Nevertheless, the regulatory relationship between IL-22 and antioxidant system in fish has not been clearly delineated. Moreover, it is uncertain whether NLRP3 is involved in the bacterial inflammatory response regulated by fish IL-22. Therefore, elucidating the regulatory role of fish IL-22 on the ROS/NLRP3 inflammasome axis and its downstream signaling responses holds profound significance for understanding host defense mechanisms and developing novel therapeutic strategies to combat microbial infections.

Identifying key target genes for resistance to *A. hydrophila* infection in fish and investigating the protective mechanisms of these genes on tissues will offer compelling evidence for breeding fish strains resistant to *A. hydrophila*. Therefore, in this study, we identified an IL-22 homologue in *M. amblycephala* and analyzed its structural features, evolutionary relationship, tissue distribution and expression pattern. Subsequently, we evaluated the protective effect of recombinant *M. amblycephala* IL-22 (rMaIL-22) protein against *A. hydrophila* infection, and further assessed the effects of rMaIL-22 on ROS/NLRP3 inflammasome axis, inflammation response, apoptosis and tissue injury in the organism. In summary, these findings establish a robust groundwork for advancing our understanding of the role of MaIL-22, particularly in regulating the ROS/NLRP3 inflammasome axis, thereby offering potential novel strategies for effectively controlling and managing aquaculture diseases.

## 2 Materials and methods

### 2.1 Ethics statement

All experimental procedures adhered to the Guide for the Care and Use of Laboratory Animals issued by the Ministry of Science and Technology in China. The Experimental Animal Ethics Committee of Huazhong Agricultural University granted approval for the protocol (approval number: HZAUFI-2022-0024). Surgical experiments were carried out under MS-222 anesthesia to minimize fish suffering in accordance with ethical standards.

### 2.2 Acquisition and sequence analysis of the *M. amblycephala* il-22 (Mail-22) gene

The *Mail-22* sequence was obtained from the *M. amblycephala* genome of the National Center for Biotechnology Information (NCBI). Subsequently, the *Mail-22* sequence was cloned and identified using specific primers (see in **Supplementary Table 1**). Meanwhile, Simple Modular Architecture Research Tool (SMART, http://smart.embl-heidelberg.de/) and Gene Structure Display Server 2.0 (GSDS 2.0, http://gsds.cbi.pku.edu.cn/) were used to predict the signal peptide and gene structure map, respectively. In addition, based on the amino acid sequences of *M. amblycephala* IL-22 and teleost IL-22s, sequence comparison of IL-22s was performed using Clustal X2 and analyzed for sequence identity.

### 2.3 Protein structure prediction and properties analysis

Protein models of *M. amblycephala* IL-22 was constructed based on the Iterative Threading Assembly Refinement (https://zhanggroup.org/I-TASSER/) (26). The protein models were visualized by PyMOL 2.5 software. The physicochemical characteristics of proteins were further analyzed using ExPASy (http://web.expasy.org/protparam/) (27).

### 2.4 Phylogenetic analysis

The amino acid sequences of 21 IL-22s from mammals, birds, amphibians and teleosts were retrieved from the NCBI database were imported into MEGA X. The above amino acid sequences were compared by the default parameters of Clustal X2. The IL-22 proteins phylogenetic tree was constructed by the Neighbor-Joining (NJ) method (28) with Dayhoff model, pairwise gap deletion, 1,000 bootstraps and visualized by iTOL (http://itol.embl.de/help.cgi) (29).

### 2.5 Expression, purification and verification of rMaIL-22 protein

rMaIL-22 protein was performed with reference to previous report (30). In brief, PCR amplification was performed using Taq DNA polymerase (CW0680L, CWBIO, Jiangsu, China). The *il-22* CDS amplified by gene-specific primer (see **Supplementary Table 1**) was inserted into pET-32a by *Eco*R I and *Xho* I. The recombinant plasmid pET-32a-IL-22 was transformed into *Escherichia coli* BL21 (DE3) Trx cells (TSC-E06, Tsingke, Beijing, China), and the TrxA-tagged rMaIL-22 protein expression was produced at 37°C for 6 h with isopropyl-β-D-thiogalactoside (IPTG, ST098-1g, Beyotime, Shanghai, China) at final concentration of 1.0 mM. rMaIL-22 protein was purified by the Protein Purification Kit (Denaturant-resistant) (P2229S, Beyotime, Shanghai, China) according to the manufacturer’s instructions. Subsequently, we validated the rMaIL-22 protein by SDS-PAGE Kit (P0012A, Beyotime, Shanghai, China) and western blot (WB) with anti-His antibody (AE003, ABclonal, Wuhan, China). rMaIL-22 was identified as a band with the correct molecular weight (38.1 kDa). Purified rMaIL-22 protein concentration was quantified using BCA kit (P0010S, Beyotime, Shanghai, China). Thioredoxin A (TrxA) protein was purified and preserved in our laboratory for control group (31).

### 2.6 Tissue expression analysis of Mail-22 and bacterial challenge

Healthy *M. amblycephala* (25 ± 5 g) were purchased from the National *M. amblycephala* farm in Ezhou, China, placed in a recirculating culture system at 28°C and fed commercial diets twice daily (04047, Haid, Guangdong, China). They were acclimated for 14 d before experiments. To detect the expression pattern of *Mail-22*, tissues (intestine, gill, head kidney, trunk kidney, spleen, blood and liver) were collected from12 healthy fish (three biological replicates, each contained four fish samples), and then quickly frozen in liquid nitrogen for RNA extraction or standby preservation at -80°C.

*A. hydrophila* was kindly donated by Associate Professor Luo Yi, College of Fisheries, Huazhong Agricultural University (HZAU) (32). The dose of *A. hydrophila* for infecting *M. amblycephala* was determined according to the methodology of a previous study (20). Briefly, 40 fish were randomly divided into 4 groups (n = 10). Fish were injected intraperitoneally (i.p.) with different concentrations of *A. hydrophila* (1.0 × 10^8^ CFU/mL, 1.0 × 10^7^ CFU/mL, and 1.0 × 10^6^ CFU/mL; 50 μL per fish) or PBS (50 μL per fish, negative control). Based on the fish survival curve, the dose was determined to be 50 μL of 1.0 × 10^7^ CFU/mL. Tissues (liver, spleen, trunk kidney and intestine) from 12 fish (three biological replicates, each contained four fish samples) were collected at each time points (0 h, 12 h, 24 h, 48 h, and 96 h) after *A. hydrophila* infection (1.0 × 10^7^ CFU/mL, 50 μL), and then quickly frozen in liquid nitrogen for RNA extraction or standby preservation at -80°C.

### 2.7 rMaIL-22 protein rescue assays

For rMaIL-22 protection rate detection, 150 fish were randomly divided into 3 groups (n = 50). In brief, fish were i.p. injected with rMaIL-22 (2 μg/g body weight) and equal dose of PBS or TrxA (negative control) in combination with previous studies on the protective effects of exogenous recombinant IL-22 (33, 34). At 12 h post protein injection, fish were challenged by i.p. injection with *A. hydrophila* (1 × 10^7^ CFU/mL, 50 μL per fish). Fish were monitored every 8 h for 7 d for typical symptoms and survival rate. Tissues (serum, liver, spleen, trunk kidney and intestine) were collected at day 2 (D2) after *A. hydrophila* injection for biological detection and histological analysis. Healthy *M. amblycephala* under the same culture conditions were used as a blank control group.

### 2.8 Antioxidant enzyme assays

The enzyme activities of malondialdehyde (MDA, A003-1), total superoxide dismutase (T-SOD, A001-1), catalase (CAT, A007-1-1), and glutathione peroxidase (GSH-PX, A005-1) in tissues (liver, spleen, trunk kidney and intestine) were assayed by commercial kits (Nanjing Jiancheng Bioengineering Institute, Nanjing, China) according to manufacturer’s instructions.

### 2.9 Quantitative reverse transcriptase PCR assays

The mRNA levels were detected by qRT-PCR according to a previous study (5). Primer information is shown in **Supplementary Table 1**. The total RNA was extracted by TRIzon Reagent (CW0580S, CWBIO, Jiangsu, China) and mRNA integrity was detected using 2% agar. Subsequently, the obtained mRNA was reverse transcribed into cDNA using the HiScript IV RT SuperMix for qPCR (R423-01, Vazym, Nanjing, China). Meanwhile, the cDNA concentration was diluted to 1 ng/μL and stored at -80°C. The qRT-PCR mixture was configured using MonAMP™ SYBR Green qPCR Mix (MQ10101S, Monad, Suzhou, China) according to the manufacturer’s instruction. The qRT-PCR reaction was performed on CFX Connect™ Real-Time System (Bio-Rad, Berkeley, USA). qRT-PCR was programed as follows: 95°C for 5 min (pre-denaturation), 40 cycles of 95°C for 10 s (denaturation), 60°C for 30 s (annealing), and 72°C for 15 s (extension). The relative mRNA expression level was calculated using the 2^-ΔΔCT^ method, and *18S rRNA* was used as an internal reference.

### 2.10 Detection of NLRP3 protein levels

Protein levels of NLRP3 in tissues (liver, spleen, trunk kidney and intestine) were examined by WB. In brief, tissues were homogenized and lysed in RIPA buffer (G2002-30ML, Servicebio, Wuhan, China) containing protease and phosphatase inhibitors. Total protein of tissues was quantified by BCA kit (P0010S, Beyotime, Shanghai, China). WB was performed in the standard fashion using 8% SDS-PAGE gel (P0012A, Beyotime, Shanghai, China) and Trans-Blot Turbo Transfer System (Bio-Rad, Hercules, CA, USA). Finally, WB bands were visualized by electrochemiluminescence reagent (ECL, RM00021P, ABclonal, Wuhan, China) and Odyssey^®^ CLx Imaging System (LI-COR Biosciences, Lincoln, NE, USA). The following antibodies were used in this experiment: anti-NLRP3 (1:1000, A24294, ABclonal, Wuhan, China) and anti-β-actin (1:100,000, AC026, ABclonal, Wuhan, China).

### 2.11 Hematoxylin-eosin staining, reactive oxygen species staining and TUNEL staining

Tissues (liver, spleen, trunk kidney and intestine) were fixed with paraformaldehyde solution (4%) and embedded in paraffin. Subsequently, the wax blocks were cut into slices (3-4 mm). Tissue slices were dewaxed and rehydrated, and stained with HE staining. Histopathologic features were analyzed with CaseViewer software (3DHistech).

Frozen sections of tissues (liver, spleen, trunk kidney and intestine) were used for ROS immunofluorescence detection according to the previous methodology (25). In short, frozen sections were rewarmed at 25°C and added to a self-fluorescence quencher for 5 min. Then, frozen sections were incubated with ROS dye solution in a dark incubator at 37°C for 30 min. The sections were washed 3 × 10 min with PBS and incubated with the DAPI (G1012, Servicebio, Wuhan, China) at 37°C for 10 min. The sections were washed 3 × 10 min with PBS and sealed with anti-fluorescence quenching sealing tablets (G1401, Servicebio, Wuhan, China). The images were acquired under a fluorescence microscope (BX63, Olympus, Tokyo, Japan). Blue indicates nuclei and red indicates ROS-positive areas.

TUNEL assay was performed on paraffin sections using the TUNEL kit (G1501, Servicebio, Wuhan, China) according to the manufacturer’s instructions. Briefly, paraffin sections were deparaffinized for proteinase K (20 μg/mL) and PBS (with 0.1% Triton X-100) repair. The sections washed 3 × 10 min with PBS and equilibrated with the equilibration buffer (10 min, 25°C). After equilibration, the sections were incubated with 50 μL of dUTP for 1 h at 37°C, and washed 3 × 10 min with PBS. The sections were then stained with DAPI (15 min, G1012, Servicebio, Wuhan, China), and the images were further analysis under a fluorescence microscope (BX63, Olympus, Tokyo, Japan). Blue indicates nuclei and green indicates apoptosis positive signals.

### 2.12 Statistical analysis

The results were presented as mean ± standard deviation (SD), and statistical analyses were executed with GraphPad Prism 9.1.0 (GraphPad Prism Software, CA, USA). Statistical analyses were performed by Student’s t-test (* indicates *p* < 0.05; ** indicates *p* < 0.01). In survival analysis, the *p*-value was computed using the Log-rank (Mantel-Cox) test (* indicates *p* < 0.05).

## 3 Results

### 3.1 MaIL-22 exhibited conserved structural features relative to other teleost IL-22s

**Figure 1:**
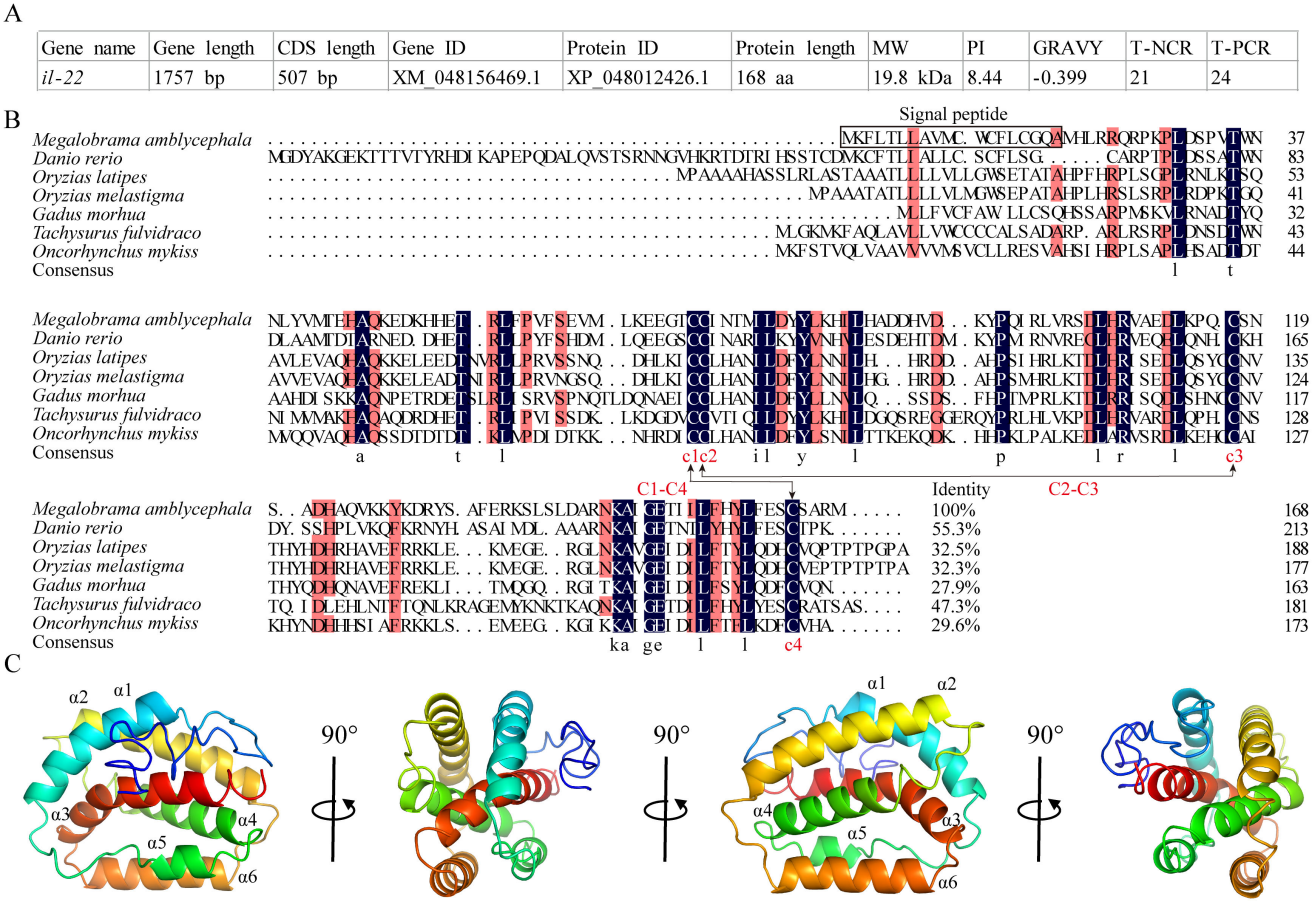
Analysis of the amino acid sequence and protein structure of MaIL-22. **(A)** Basic information of MaIL-22. MW, molecular weight; PI, isoelectric point; GRAVY, grand average of hydropathicity; T-NCR, total number of negatively charged residues (Asp + Glu); T-PCR, total number of positively charged residues (Arg + Lys). **(B)** Amino acid sequence analysis of teleost IL-22. The amino acid sequence alignment was performed by DNAMAN. Black background represents identical amino acid residues; red background represents conserved amino acid residues; dot represents gaps introduced for alignment purposes; C-C stands for disulfide bond. The NCBI accession numbers of protein used in multiple sequence analysis were as follows: *Megalobrama amblycephala*, XP_048012426.1; *Danio rerio*, BAD72867.1; *Ctenopharyngodon idella*, QNO10650.1; *Oryzias latipes*, BCB16984; *Oryzias melastigma*, XP_036066245; *Gadus morhua*, CAR63747.1; *Tachysurus fulvidraco*, AWU48777; *Oncorhynchus mykiss*, CAO02398.1. **(C)** Protein structure of MaIL-22. The secondary structures were visualized in PyMOL software. In secondary structures, the α-helices and loops are visualized in different colors.

The CDS of MaIL-22 was predicted to be 507 bp long (NCBI accession number: XM_048156469.1), encoding 168 amino acids (aa) with a molecular weight of 19.8 kDa (NCBI accession number: XP_048012426.1) (**Figure 1A**). Meanwhile, MaIL-22 protein exhibited negative grand average of hydropathicity (GRAVY) values, indicating its hydrophilic nature (**Figure 1A**). Additionally, the MaIL-22 protein contained a predicted signal peptide (1-19 aa) (**Figure 1B**). Through multiple sequence comparisons of teleost IL-22, we observed that MaIL-22 showed low identity with known homologs, generally ranging from 29.6% (*Oncorhynchus mykiss*) to 55.3% (*Danio rerio*) (**Figure 1B**). Notably, four cysteine (Cys) residues were predicted to form two intramolecular disulfide bonds (C1-C4 and C2-C3), which were conserved in teleost IL-22 (**Figure 1B**). Additionally, the secondary structure of the MaIL-22 protein comprised six α-helices (α1-α6) and loops (**Figure 1C**).

**Figure 2:**
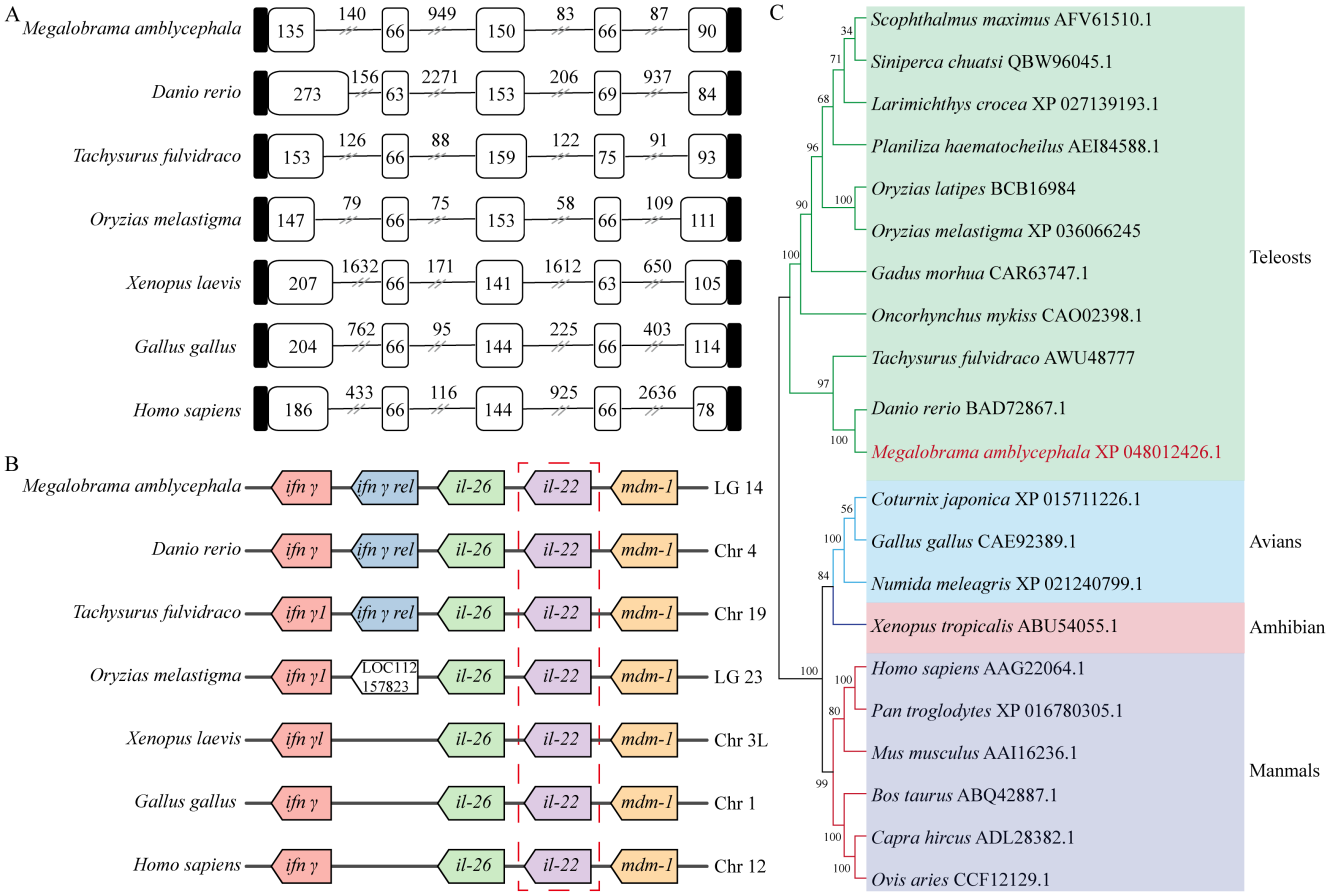
Evolutionary analysis of MaIL-22. **(A)** The comparison of IL-22 gene organization in different species. Black boxes, white boxes, and black lines indicate untranslated regions, exons, and introns, respectively. **(B)** The gene synteny of IL-22 in different species. Boxes of the same color represent orthologous genes between species. **(C)** The phylogenetic tree of IL-22. The selected amino acids were bootstrapped with 1,000 bootstrap replicates by the Neighbor-Joining (NJ) method using the MEGA 7 software. The final tree was visualized by iTOL. Different species are marked with different colored backgrounds.

The DNA sequence of MaIL-22 contained five exons and four introns, which were conserved among vertebrates. Amone them, except for the first exon, the lengths of the remaining four exons were similar among vertebrates. However, intron lengths of the *il-22* varied considerably among vertebrates (**Figure 2A**). According to our syntenic analysis (**Figure 2B**), the synteny arrangements of the *il-22* were highly conserved across teleosts. In addition, the *il-26* and *mdm-1* loci around *il-22* were conserved in vertebrates. Meanwhile, *ifn-γ rel* was only present in teleosts (**Figure 2B**). The results of the phylogenetic tree showed that MaIL-22 clustered with other teleosts IL-22 (**Figure 2C**), further confirming the reliability of MaIL-22 identification.

### 3.2 MaIL-22 was involved in the immune response of *M. amblycephala* after *A. hydrophila* infection *in vivo*

**Figure 3:**
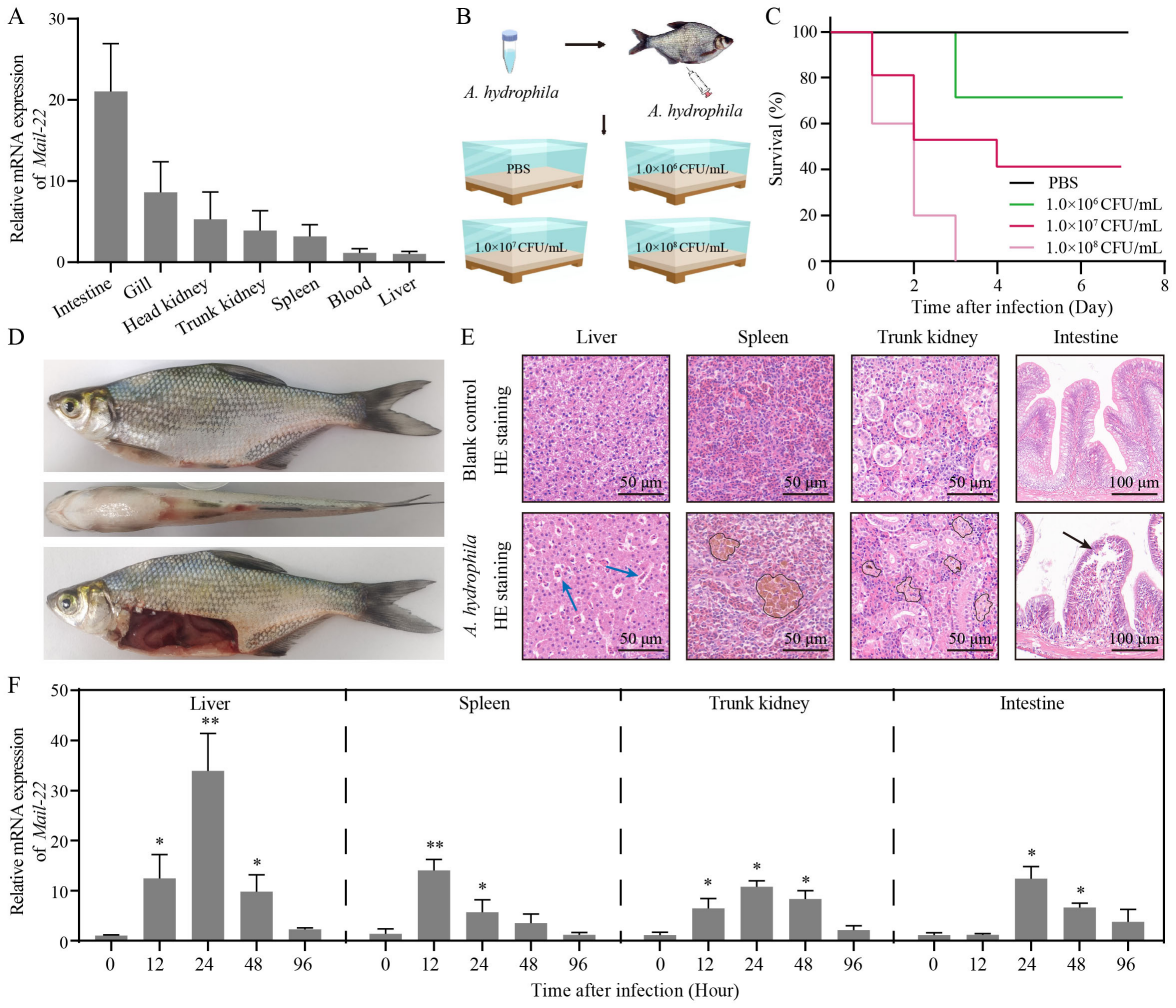
MaIL-22 was involved in the immune response of *M. amblycephala* after *A. hydrophila* infection. **(A)** MaIL-22 mRNA was detected in the seven tissues (intestine, gill, head kidney, trunk kidney, spleen, blood and liver) from healthy *M. amblycephala* by qRT-PCR. The *18S rRNA* gene was used as the internal reference. **(B)** Schematic diagram of the screening concentration of *A. hydrophila* in infected M. amblycephala. Fish were injected i.p. with different concentrations of *A. hydrophila* (1.0 × 10^8^ CFU/mL, 1.0 × 10^7^ CFU/mL, and 1.0 × 10^6^ CFU/mL; 50 μL per fish) and PBS (50 μL per fish, negative control). Fish were then observed in water tanks (28°C). **(C)** Fish survival rate was monitored and calculated after infected with different concentrations of *A. hydrophila* for 7 d (n = 10). **(D)** Primary phenotype of dead *M. amblycephala* after *A. hydrophila* infection (1.0 × 10^7^ CFU/mL, 50 μL). **(E)** HE-stained histological sections of tissues (liver, spleen, trunk kidney, and intestine) from healthy (blank control) and *A. hydrophila* infected (1.0 × 10^7^ CFU/mL, 50 μL) fish. Blue arrow represents erythrocyte infiltration. Black circle represents hemosiderin deposition. Black arrow represents villous injury. **(F)** MaIL-22 mRNA in tissues (liver, spleen, trunk kidney, and intestine) of *M. amblycephala* was examined by qRT-PCR at different time points (0 h, 12 h, 24 h, 48 h, and 96 h) after *A. hydrophila* infection. Each time contained three biological replicates (each biological replicate contained four samples). Data are represented as mean ± SD. Experimental data are analyzed using the Student’s t-test. * indicates significant differences between the *A. hydrophila*-infected group and the PBS group at corresponding time points (PBS group data not shown. * indicates *p* < 0.05; ** indicates *p* < 0.01).

We verified the constitutive expression of MaIL-22 in seven detected tissues (intestine, gill, head kidney, trunk kidney, spleen, blood and liver) of healthy *M. amblycephala* using qRT-PCR. Among them, the expression level of *Mail-22* mRNA was the highest in intestine (**Figure 3A**). To determine the appropriate infective dose of *A. hydrophila* for *M. amblycephala*, we administered different concentrations of *A. hydrophila* (1.0 × 10^8^ CFU/mL, 1.0 × 10^7^ CFU/mL, and 1.0 × 10^6^ CFU/mL; 50 μL per fish) via intraperitoneal injection, alongside a control group injected with PBS (50 μL per fish) (**Figure 3B**). Dose-response experiments revealed that 1.0 × 10^7^ CFU/mL (50 μL per fish) resulted in a gradient mortality of *M. amblycephala* with a certain sample size retained (**Figure 3C**). Concurrently, fish infected with *A. hydrophila* (1.0 × 10^7^ CFU/mL) exhibited abdominal swelling and redness in visceral tissues (**Figure 3D**). Subsequently, after observing symptoms, we conducted histopathological analysis of the visceral tissues (liver, spleen, trunk kidney, and intestine) using HE staining. Our findings demonstrated that *A. hydrophila* infection caused erythrocyte infiltration in liver, hemosiderin deposition in spleen and trunk kidney, and villous injury in intestine compared to healthy *M. amblycephala* (**Figure 3E**). To determine whether MaIL-22 was involved in anti-bacterial immunity, we examined changes in *Mail-22* mRNA expression levels in visceral tissues. After *A. hydrophila* infection, *Mail-22* mRNA was significantly dynamically changed in visceral tissues (**Figure 3F**). These results suggested that MaIL-22 was involved in the immune response after *A. hydrophila* infection *in vivo*.

### 3.3 rMaIL-22 effectively enhanced resistance to *A. hydrophila* in *M. amblycephala*

**Figure 4:**
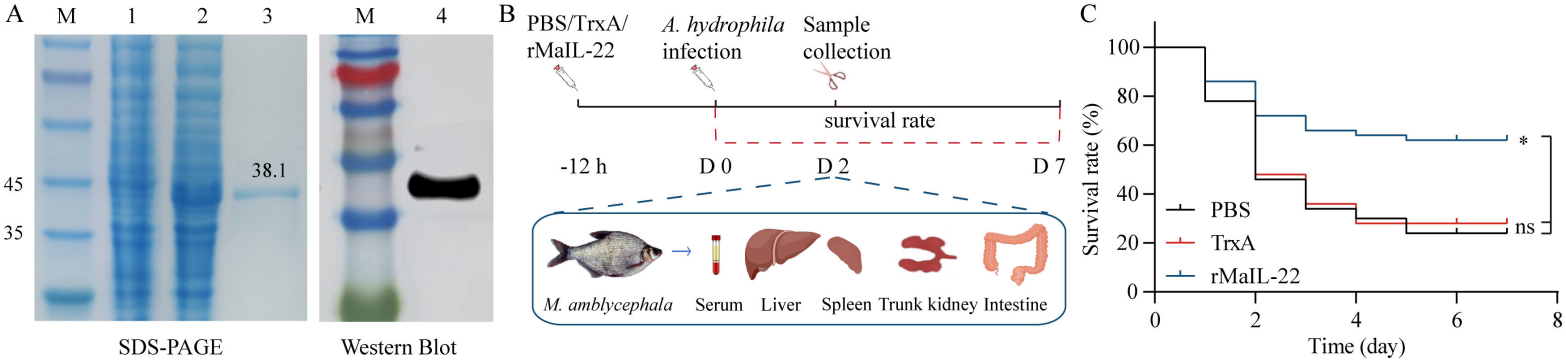
Recombinant MaIL-22 (rMaIL-22) effectively enhances resistance to *A. hydrophila* in M. amblycephala. **(A)** SDS-PAGE and WB analyses of rMaIL-22. Lane M, protein marker; lane 1, not induced with IPTG; lane 2, induced with IPTG; lane 3, purified rMaIL-22; lane 4, The rMaIL-22 was confirmed by WB with anti-His tag Ab. **(B)** Schematic diagram of the animal experimental protocol for assessing the protective effect of rMaIL-22. *M. amblycephala* were injected i.p. with rMaIL-22 (2 μg/g body weight) or isodose PBS/TrxA (as negative control), followed by a single dose of *A. hydrophila* (50 μL, 1.0 × 10^7^ CFU/mL) after 12 h stimulation. At D2 after *A. hydrophila* infection, samples (serum, liver, spleen, trunk kidney and intestine) were collected for subsequent testing. **(C)** Survival rate was monitored and calculated for 7 d (n = 50). In survival analysis, the *p*-value is computed using the Log-rank (Mantel-Cox) test (* indicates *p* < 0.05).

To further gain insight into the biological function of MaIL-22, we obtained rMaIL-22 using a prokaryotic expression system. As shown in **Figure 4A**, SDS-PAGE and WB confirmed the successful production of rMaIL-22 (38.1 kDa). To further test the protective effect of rMaIL-22 *in vivo*, we performed an infection experiment and monitored survival rates over a period of 7 days. Fish were infected with *A. hydrophila* through i.p. after 12 h of treatment with either rMaIL-22, TrxA or PBS (**Figure 4B**). It can be seen that different groups of *M. amblycephala* showed a watershed in the number of deaths on D2 after infection with *A. hydrophila* (**Figure 4C**). Therefore, we collected *M. amblycephala* tissues (serum, liver, spleen, trunk kidney and intestine) at this time point for subsequent testing (**Figure 4B**). Furthermore, the survival rates of the groups with PBS (24%) and TrxA (28%) were not significantly different. Importantly, the rMaIL-22 group (62%) had a significantly higher survival rate than the PBS and TrxA groups, implying that rMaIL-22 was effective in enhancing the resistance of *M. amblycephala* to *A. hydrophila*.

### 3.4 rMaIL-22 significantly inhibited *A. hydrophila*-induced activation of the ROS/NLRP3 inflammasome axis

**Figure 5:**
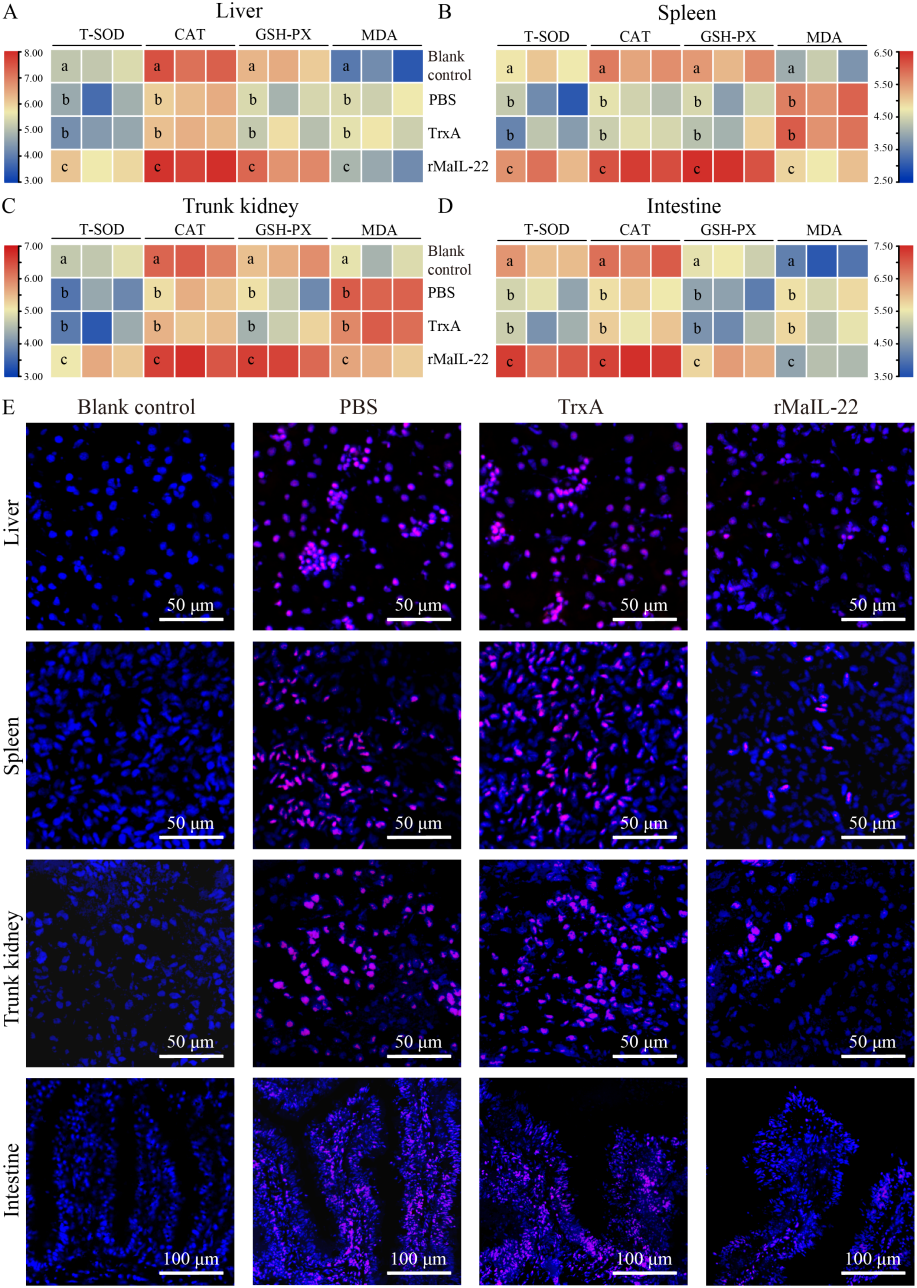
Effect of rMaIL-22 on the antioxidant capacity of *M. amblycephala* after *A. hydrophila* infection. The liver **(A)**, spleen **(B)**, trunk kidney **(C)** and intestine **(D)** antioxidant capacity enzymes (T-SOD, CAT, GSH-PX and MDA) were measured by the corresponding specific commercial kits (Nanjing Jiancheng Bioengineering Institute, Nanjing, China). Each group contained three biological replicates (each biological replicate contained four samples). Significant differences (*p* < 0.05) between groups are indicated by different superscript letters (a, b, and c). **(E)** Representative image of tissues (liver, spleen, trunk kidney, and intestine) ROS staining. Blue indicates nuclei and red indicates ROS-positive areas.

To further explore the protective pathways of rMaIL-22 on *M. amblycephala*, we examined the antioxidant system and ROS accumulation in different tissues (liver, spleen, trunk kidney and intestine). After *A. hydrophila* infection, antioxidant enzyme (T-SOD, CAT and GSH-PX) activities showed a decreasing trend in the PBS and TrxA groups and were significantly lower than in the blank control group (**Figures 5A–D**). In contrast, the rMaIL-22-treated group showed enhanced antioxidant enzyme activity in tissues and was significantly higher than the PBS and TrxA groups (**Figures 5A–D**). Concurrently, the index of oxidative injury (MDA) was significantly higher in the PBS and TrxA groups than in the rMaIL-22 group (**Figures 5A–D**). In addition, ROS signaling was reduced in the rMaIL-22 group relative to the PBS and TrxA groups (**Figure 5E**). This suggested that *A. hydrophila* disrupted the antioxidant system of *M. amblycephala* and exacerbated the accumulation of ROS in tissues, whereas rMaIL-22 enhanced the ability of the tissues to scavenge ROS by activating the antioxidant enzyme activity.

**Figure 6:**
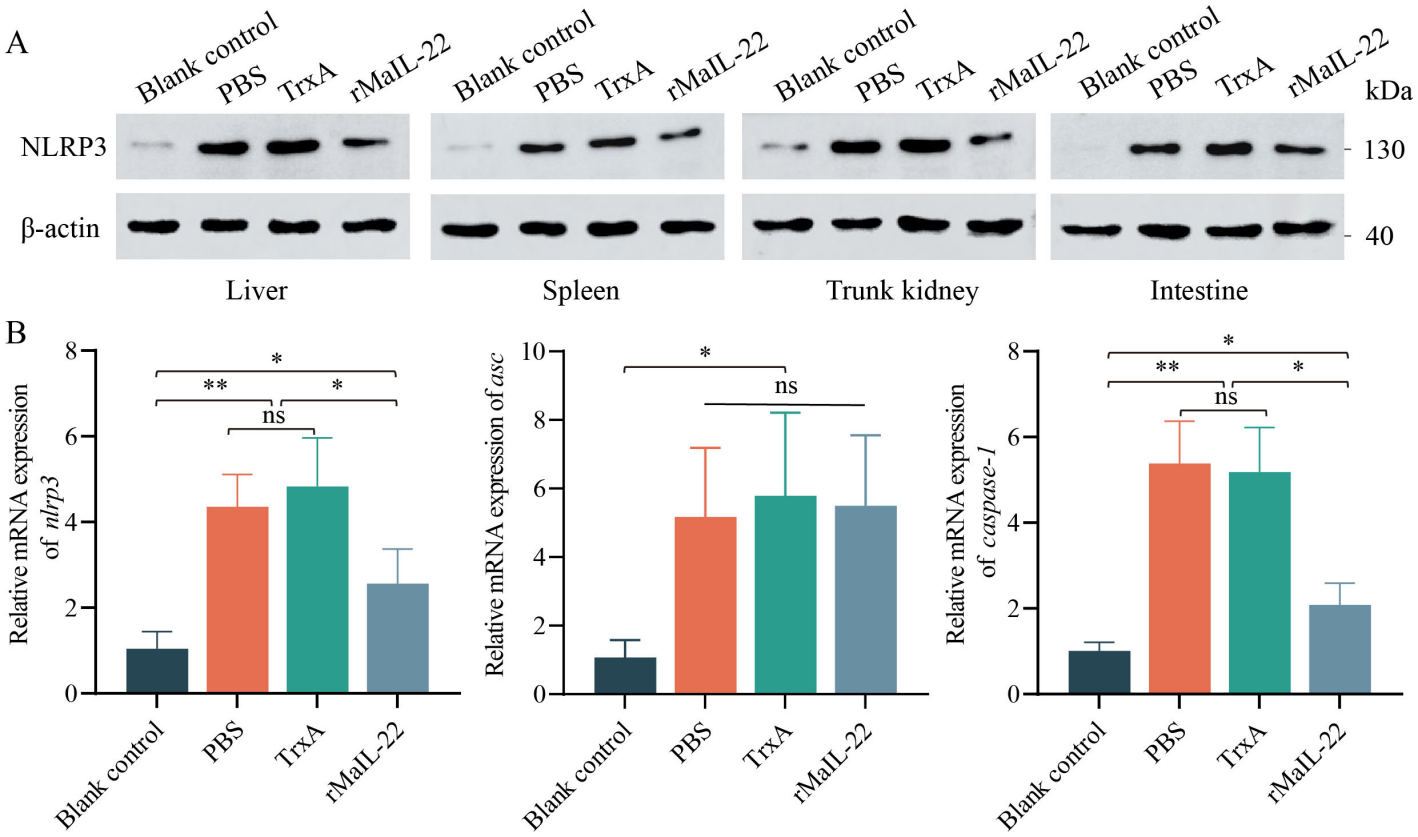
Effect of rMaIL-22 on NLRP3 inflammasome (*NLRP3*/*ASC*/*Caspase-1*) signaling pathway activity in *M. amblycephala* after *A. hydrophila* injection. **(A)** Changes in NLRP3 protein levels in visceral tissues (liver, spleen, trunk kidney and intestine) under different treatments detected by WB with anti-NLRP3 and anti-β-actin Ab (as the internal control). **(B)** The *nlrp3, asc and caspase-1* mRNA expression in serum by qRT-PCR. The *18S rRNA* gene was used as a reference gene. Each group contained three biological replicates (each biological replicate contained four samples). Data are represented as mean ± SD. ns means no significance. *p < 0.05, **p < 0.01.

Considering the pivotal role of ROS in NLRP3 inflammasome activation, we sought to understand whether rMaIL-22 can inhibit NLRP3 inflammasome activation induced by *A. hydrophila* while scavenging ROS. In **Figure 6A**, NLRP3 protein levels were higher in the PBS and TrxA groups than in blank control group after *A. hydrophila* infection. However, NLRP3 protein levels were reduced in the rMaIL-22 group relative to the PBS and TrxA groups. Serum cytokine expression levels reflect overall immune activity. Therefore, we examined changes in mRNA levels of NLRP3 inflammasome components (*nlrp3*, *asc* and *caspase-1*) in serum (**Figure 6B**). The mRNA levels of NLRP3 inflammasome components were significantly higher in both the PBS and TrxA groups compared to the blank control group. Meanwhile, there was no significant difference between the PBS and TrxA groups. Moreover, rMaIL-22 treatment reduced the mRNA levels of *nlrp3* and *caspase-1* but did not significantly affect the mRNA expression levels of *asc* relative to the PBS and TrxA groups. This indicated that rMaIL-22 may hinder the assembly of NLRP3 inflammasome mainly by inhibiting NLRP3 synthesis. Taken together, MaIL-22 may help attenuate *A. hydrophila*-induced activation of the ROS/NLRP3 inflammasome axis.

### 3.5 rMaIL-22 effectively inhibited inflammation, reduced apoptosis, and attenuated tissue injury

**Figure 7:**
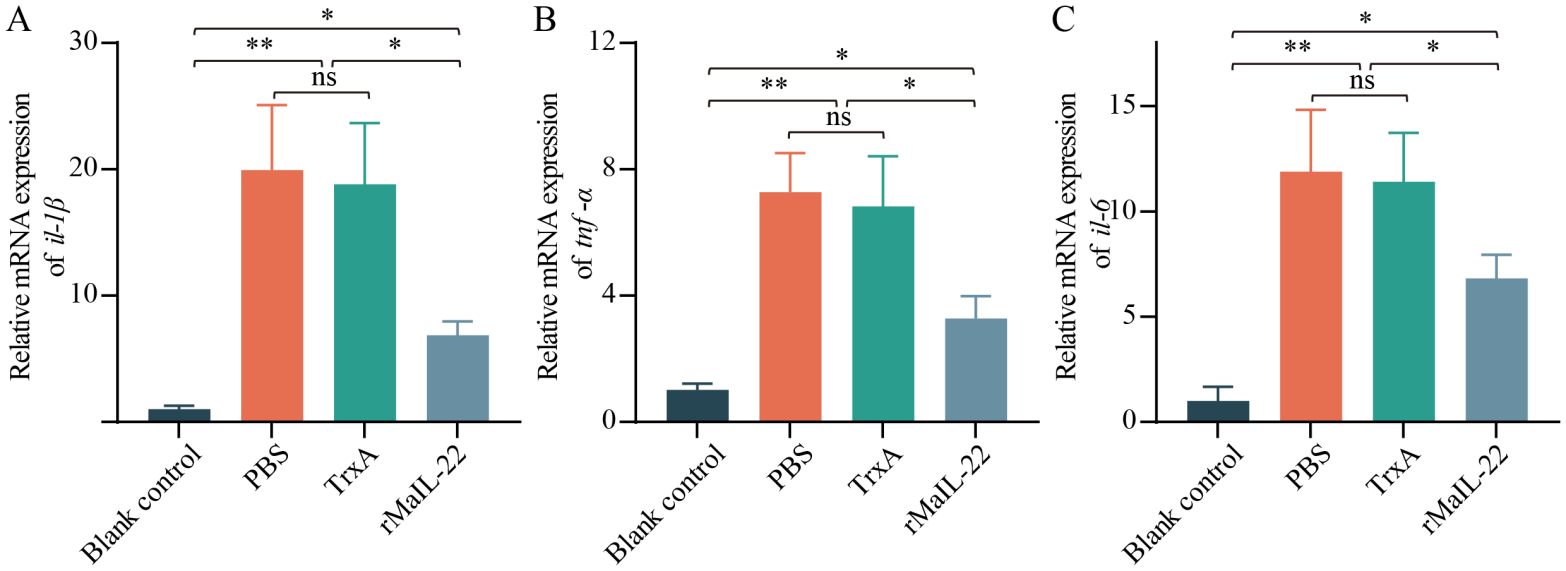
mRNA expression patterns of representative pro-inflammatory genes in serum. The expressions of *il-1β* **(A)**, *tnf-α* **(B)** and *il-6* **(C)** mRNAs were detected at D2 after *A. hydrophila* infection by qRT-PCR. *18S rRNA* was used a reference gene. Each group contained three biological replicates (each biological replicate contained four samples). Data are represented as mean ± SD. ns means no significance. *p < 0.05, **p < 0.01.

**Figure 8:**
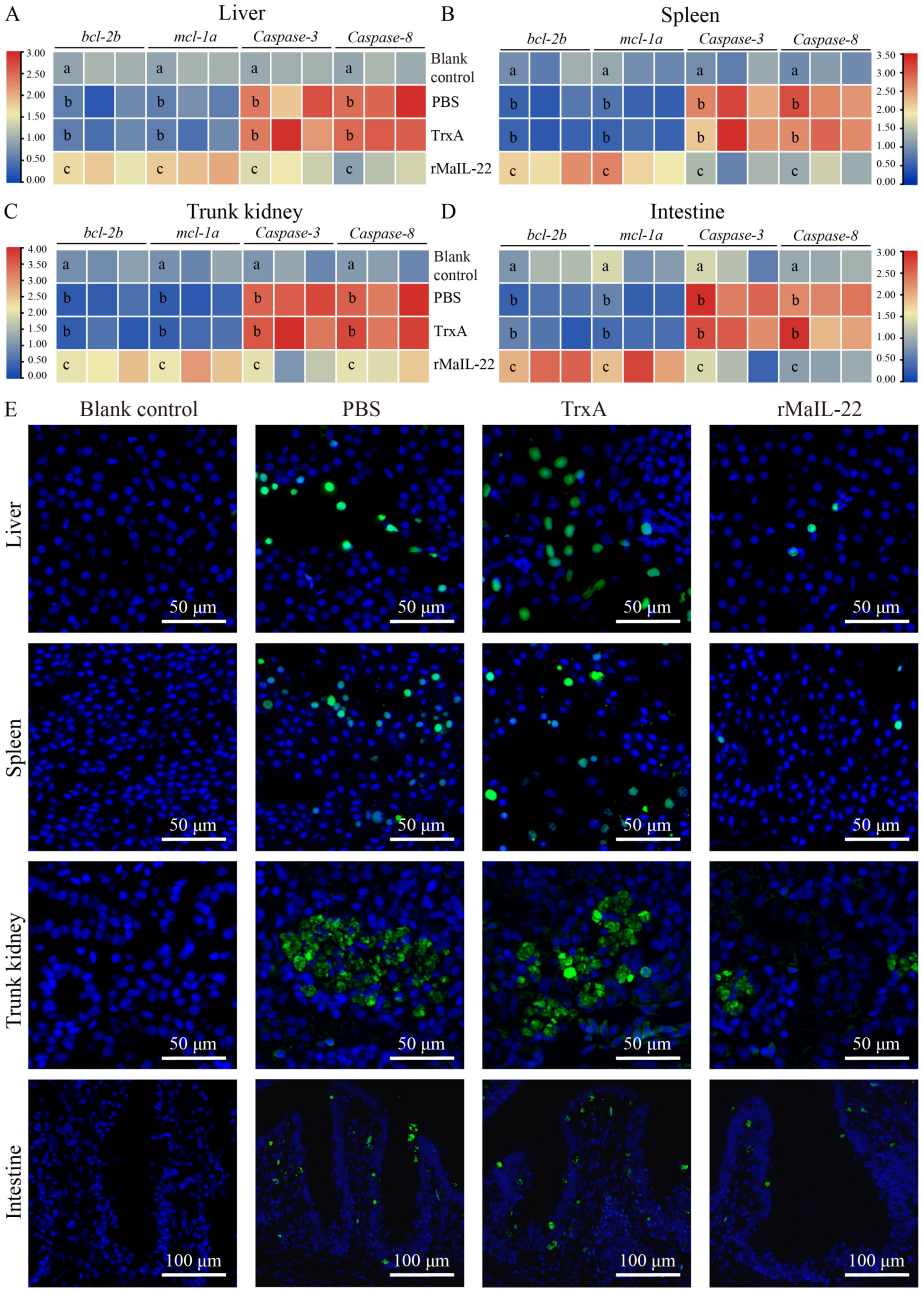
Effect of rMaIL-22 on the antiapoptotic capacity of *M. amblycephala* after *A. hydrophila* infection. The liver **(A)**, spleen **(B)**, trunk kidney **(C)** and intestine **(D)** apoptosis-related mRNA expression (*bcl-2b*, *mcl-1a*, *caspase-3* and *caspase-8*) were measured by qRT-PCR. The *18S rRNA* gene was used as a reference gene. Each group contained three biological replicates (each biological replicate contained four samples). Significant differences (*p* < 0.05) between groups are indicated by different superscript letters (a, b, and c). **(E)** Representative images of tissues (liver, spleen, trunk kidney, and intestine) TUNEL staining. Blue indicates nuclei and green indicates apoptotic cell.

**Figure 9:**
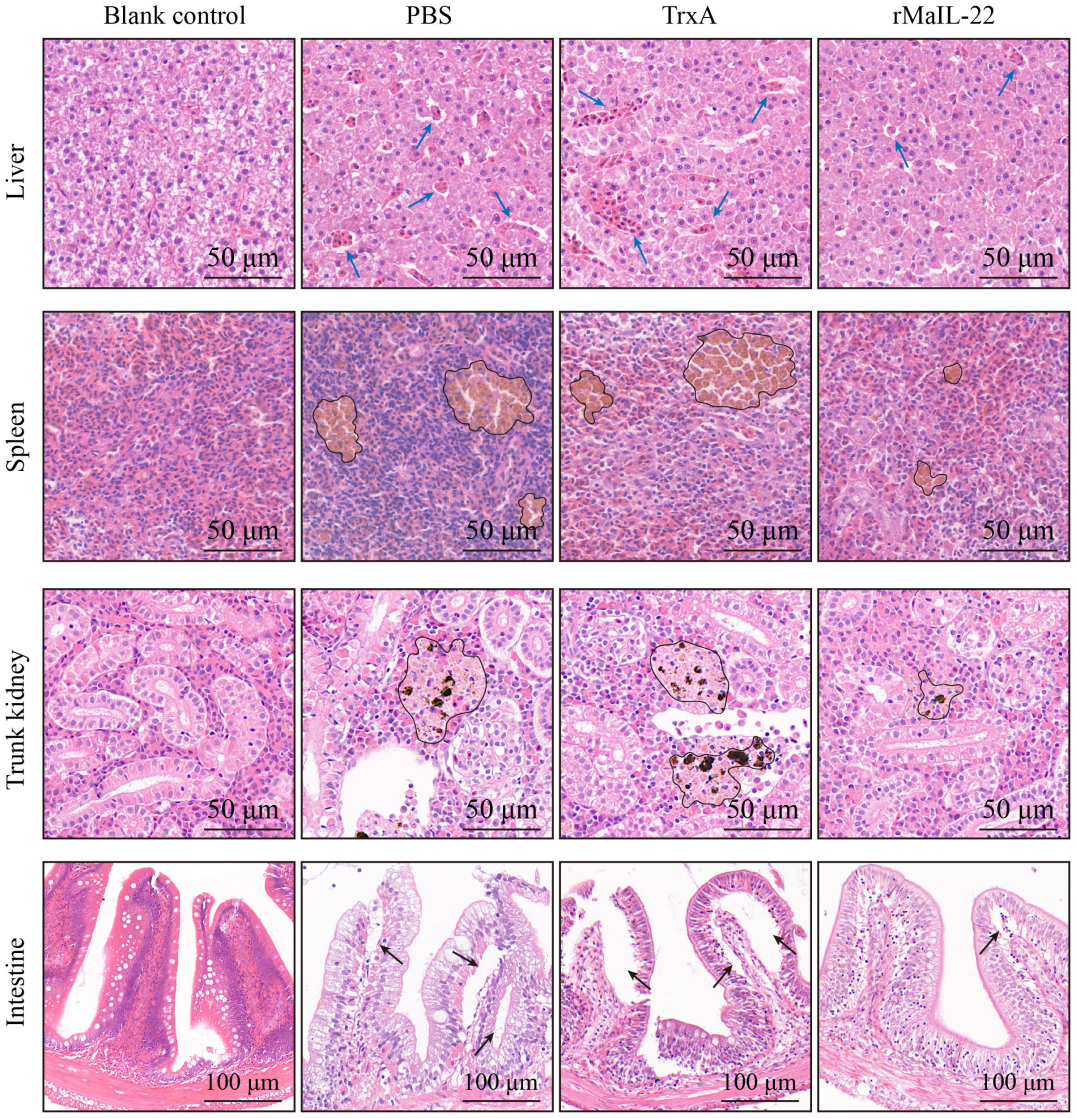
Detection of histopathological injury in *M. amblycephala* after *A. hydrophila* infection. Representative images of visceral tissues (liver, spleen, trunk kidney and intestine) with HE staining. Tissues were collected at D2 post *A. hydrophila* infection. Blue arrow represents erythrocyte infiltration. Black circle represents hemosiderin deposition. Black arrow represents villous injury.

Given that the activation of ROS/NLRP3 inflammasome axis further induces inflammation and apoptosis, we further investigated the expression of inflammatory factors and apoptosis *in vivo*. Compared with the blank control group, the mRNA levels of pro-inflammatory factors (*il-1β*, *tnf-α* and *il-6*) were significantly up-regulated in the PBS and TrxA groups. Meanwhile, the mRNA levels of pro-inflammatory factors were significantly lower in the rMaIL-22 group than in the PBS and TrxA groups (**Figures 7A–C**). This implied that rMaIL-22 contributed to the inhibition of the inflammatory response triggered by *A. hydrophila*. Subsequently, we examined the mRNA expression of representative apoptotic genes in different tissues (liver, spleen, trunk kidney and intestine) by qRT-PCR. The transcriptional levels of anti-apoptotic genes (*bcl-2b* and *mcl-1a*) exhibited a significant increase, while the expression of pro-apoptotic genes (*caspase-3* and *caspase-8*) demonstrated a significant decrease following the rMaIL-22 group compared with the PBS and TrxA groups (**Figures 8A–D**). TUNEL staining results showed a significant decreased in TUNEL-positive cells in the rMaIL-22 group compared with the PBS and TrxA groups (**Figure 8E**), suggesting that MaIL-22 might contribute to attenuate apoptosis induced by *A. hydrophila*. To further assess the protective effect of MaIL-22, we performed pathological analyses of the tested tissues (**Figure 9**). The tissues in the blank control group showed normal cellular morphology with no visible lesions. After *A. hydrophila* infection, there were no significant differences in pathological features between the PBS and TrxA groups. Importantly, treatment with rMaIL-22 attenuated liver erythrocyte infiltration, spleen and trunk kidney hemosiderin deposition, and intestinal villi injury relative to the PBS and TrxA groups. These results collectively underscored the crucial protective role of rMaIL-22 *in vivo*.

## 4 Discussion

IL-22 plays a crucial role in maintaining immune system balance and is key participant in the defense against pathogenic microbial infections (35). Understanding the functions of IL-22 is vital for the prevention and treatment of diseases. Despite their significance in fish immune system, there has been a limited focus on the systematic analysis of the fish IL-22 in existing research. In this study, our objective was to enhance the understanding of the fish IL-22 by identifying and analyzing MaIL-22. We further characterized MaIL-22 by structural characteristic, evolutionary relationship, tissue distribution and expression pattern. Moreover, we investigated the role of rMaIL-22 in conferring resistance to *A. hydrophila* infection in *M. amblycephala*. This study contributes valuable insights to the broader understanding of antimicrobial immune responses of fish IL-22.

In this investigation, we successfully characterized the CDS of MaIL-22. The predicted CDS length is 507 bp, encoding a protein comprising 168 aa with a molecular mass of 19.8 kDa. This size aligns with the typical range observed in members of the teleost IL-22 family (16). Notably, the MaIL-22 protein exhibited hydrophilic properties, potentially impacting its solubility and interactions with other intracellular components (36). Moreover, the presence of a signal peptide within the N-terminal region suggests a secretory role for MaIL-22, aligning with the structural characteristics previously reported for IL-22 in other teleosts (10, 37). Interestingly, we observed low sequence homology of MaIL-22 with other teleost, which may reflect species-specific adaptations and evolutionary differences in the immune response mechanisms of teleost fish. These differences could be due to varying pathogen encounters and selective pressures on their immune systems (38). Importantly, the conservation of four Cys residues in teleost, and these residues are predicted to form two intramolecular disulfide bonds (Cys^73^-Cys^165^ and Cys^74^-Cys^118^). These conserved elements are pivotal for the protein’s proper folding, stability, and biological activity, suggesting evolutionary constraints may have preserved these structural features to maintain functional integrity of IL-22 (39, 40). Additionally, the secondary structure of MaIL-22, composed of six α-helices, is consistent with the results shown for the crystalline architecture of *D. rerio* IL-22 (40). This structure may be crucial for the protein’s ability to bind to its receptor and initiate downstream signaling pathways (41).

Intron-exon structure is important for understanding gene conservation during evolution (42). Despite the distinctive evolutionary pattern observed in teleosts, the gene structure of many genes closely mirrors that of other higher vertebrates (43–45). The gene structure of MaIL-22, comprising five exons and four introns, is conserved among vertebrates. This conservation may suggest that the *IL-22* gene has undergone purifying selection to maintain its functional integrity throughout vertebrate evolution (46). The similarity in *IL-22* exon lengths among vertebrates, barring the first exon, indicates that these regions may play critical roles in the protein’s structure and function. Conversely, variable intron lengths may indicate adaptability to species-specific regulatory demands without compromising protein-coding function (46). Syntenic analysis highlights the evolutionary preservation of genomic regions surrounding *il-22* (e.g., *il-26* and *mdm-1*), possibly due to functional constraints or selective pressures maintaining gene organization (38). Notably, the exclusive presence of *ifn-γ rel* in teleosts suggests a species-specific adaptation or divergence in immune response mechanisms, warranting further investigation. Phylogenetic analysis placing MaIL-22 within the teleosts IL-22 clade provides robust evidence for its homology and evolutionary relatedness to counterparts in other species. This clustering affirms MaIL-22 identification reliability and implies a shared evolutionary history and potentially similar functional roles among teleosts.

Gene expression patterns are important clues to explore their physiological functions (1). *il-22* mRNA was detected in different types of tissues in teleosts, such as immune tissues (e.g. body kidneys, spleen and liver) and non-immune tissues (e.g. muscle, brain and heart) (12, 13, 47). Our study found that healthy *M. amblycephala* had constitutive expression of *il-22* in most immune-related tissues. Meanwhile, *Mail-22* mRNA expression was highest in the intestine among the tested tissues, which is consistent with previous studies (12, 47). Importantly, *Mail-22* mRNA was significantly upregulated in visceral tissues following *A. hydrophila* infection. This means that Mail-22 is not only involved in mucosal immunity, but also in non-mucosal tissue immunity. Furthermore, these results suggest that MaIL-22 may play an important role in the regulation of *A. hydrophila* infection.

Protective rate serves as the most accurate reflection of a protein’s protective function. *M. amblycephala* treated with rMaIL-22 showed enhanced resistance to *A. hydrophila* infection. This observation appears to be attributed to the potent antioxidant and anti-inflammatory capacities of rMaIL-22. When fish are infected with bacteria, excess ROS are inevitably generated, potentially causing inflammation response and tissue injury (20). In response, fish can activate their enzymatic antioxidant systems (such as T-SOD, CAT, and GSH-PX) to minimize tissue injury and inflammatory responses caused by excess ROS (48). Among them, the augmentation of CAT and T-SOD activity has been extensively reported to reduce bacterial load and mitigate injury in farmed fish tissues (7, 20, 49). Therefore, potent antioxidant activity plays an effective role in fish defense against bacterial infection (49). In our study, we observed a decrease in ROS levels concurrent with enhanced CAT and SOD activities in the rMaIL-22-treated group compared with the PBS and TrxA groups. This implied that rMaIL-22 enhanced anti-oxidant defense and ROS scavenging ability in *M. amblycephal*. Therefore, our findings further confirm that IL-22 is an essential target for the regulation of the antioxidant system in fish. Meanwhile, the antioxidant function of rMaIL-22 may play an important role in attenuating *A. hydrophila*-induced tissues injury.

NLRP3 inflammasome, as a ROS downstream sensor, is essential in the inflammatory response (50). NLRP3 inflammasome can promote IL-1β secretion by activating Caspase-1, thereby leading to inflammation (22). Lipopolysaccharide (LPS) significantly induces elevated levels of NLRP3 inflammasome (NLRP3, Casepase-1 and ASC) proteins and mRNAs in the liver and intestine of common carp (*Cyprinus carpio*) (51). In the present study, *A. hydrophila* induced up-regulation of NLRP3 protein levels in visceral tissues and NLRP3 inflammasome mRNA levels in serum. This suggests that NLRP3 inflammasome is involved in bacterial diseases in teleosts. Meanwhile, we found that rMaIL-22 treatment inhibited NLRP3 protein synthesis in the viscera. Differently, relative to the PBS and TrxA groups, rMaIL-22 treatment did not significantly reduce *asc* mRNA expression levels in serum. In conjunction with previous studies, IL-22 has been shown to inhibit the activation of the NLRP3 inflammasome by suppressing the synthesis of NLRP3 and Caspase-1 proteins, thereby attenuating mammalian bacterial diseases and tissue injury (52, 53). This highlights that NLRP3 and Caspase-1, but not ASC, are key targets for IL-22 to inhibit NLRP3 inflammasome activation. Therefore, we speculate that NLRP3 and Caspase-1 may be key targets for IL-22 inhibition of *A. hydrophila*-induced NLRP3 inflammasome activation in fish.

Activation of the ROS/NLRP3 inflammasome axis may further induce oxidative stress and subsequently trigger an inflammatory response and apoptosis (54, 55). Previous studies have shown that inhibition of ROS/NLRP3 inflammasome activation attenuates splenic inflammatory injury in carp by suppressing pro-inflammatory factor (*il-1β*, *il-6* and *tnf-α*) secretion (56). IL-1β, IL-6 and TNF-α, as important pro-inflammatory response indicators, reflect the degree of organismal pathology (57). In the present study, the significant down-regulation of pro-inflammatory cytokines (*il-1β*, *il-6* and *tnf-α*) in the rMaIL-22 group compared to the PBS and TrxA groups underscores the potent anti-inflammatory effect of rMaIL-22. This observation is particularly noteworthy given the established link between the activation of the ROS/NLRP3 inflammasome axis and the subsequent release of these cytokines, which are pivotal in driving inflammation and tissue injury. The ability of rMaIL-22 to inhibit the expression of these pro-inflammatory factors suggests that it may disrupt the activation of this axis and thereby exert an anti-inflammatory effect. Moreover, excessive ROS-induced apoptosis can lead to tissue injury and impair the host’s ability to mount an effective immune response (58). *bcl-2* and *mcl-1* are antiapoptotic BCL-2 family members with superior apoptosis inhibitory capacity (59). Meanwhile, *caspase-3* and *caspase-8* are considered key regulators of apoptosis induction (60). IL-22 can alter the expression of anti-apoptotic (such as *bcl-2* and *mcl-1*) and pro-apoptotic factors (such as *caspase-3* and *caspase-8*) by clearing ROS and inhibiting the secretion of pro-inflammatory factors (*il-1β*, *il-6* and *tnf-α*), thereby reducing apoptosis (24, 61), which was further confirmed in our study. Furthermore, we provide evidence that IL-22 regulates bacterial-induced apoptosis in teleosts.

The pathological state of the tissue further validates the protective efficacy of the protein (62). Therefore, we investigated the extent of pathological injury to the main injury d tissues (liver, spleen, somatic kidney and intestine) of *A. hydrophila* in *M. amblycephala*. As anticipated, the tissues treated with rMaIL-22 exhibited mild tissue injury compared with the PBS and TrxA group. The reason may be that rMaIL-22 treatment increased the antioxidant capacity of *M. amblycephala* and inhibited further injury responses induced by *A. hydrophila*. This suggests that MaIL-22 contributes to maintain the normal morphology of tissues and hence enhances the organism’s resistance to pathogenic bacteria. Furthermore, in mammals, spleen *IL-22* mRNA levels have been reported to be significantly elevated and activate disease resistance after bacterial and viral infections (63, 64). Meanwhile, IL-22 serves as a pivotal mediator of host mucosal immunity, possessing functions such as apoptosis inhibition, alleviation of intestinal inflammation, and tissue injury restoration in colitis (65, 66). These findings suggest that IL-22 may play a protective role in different species. Consequently, IL-22 may be a potential target for lowering pathogenic microbial infections and eliminating inflammation.

## 5 Conclusion

**Figure 10:**
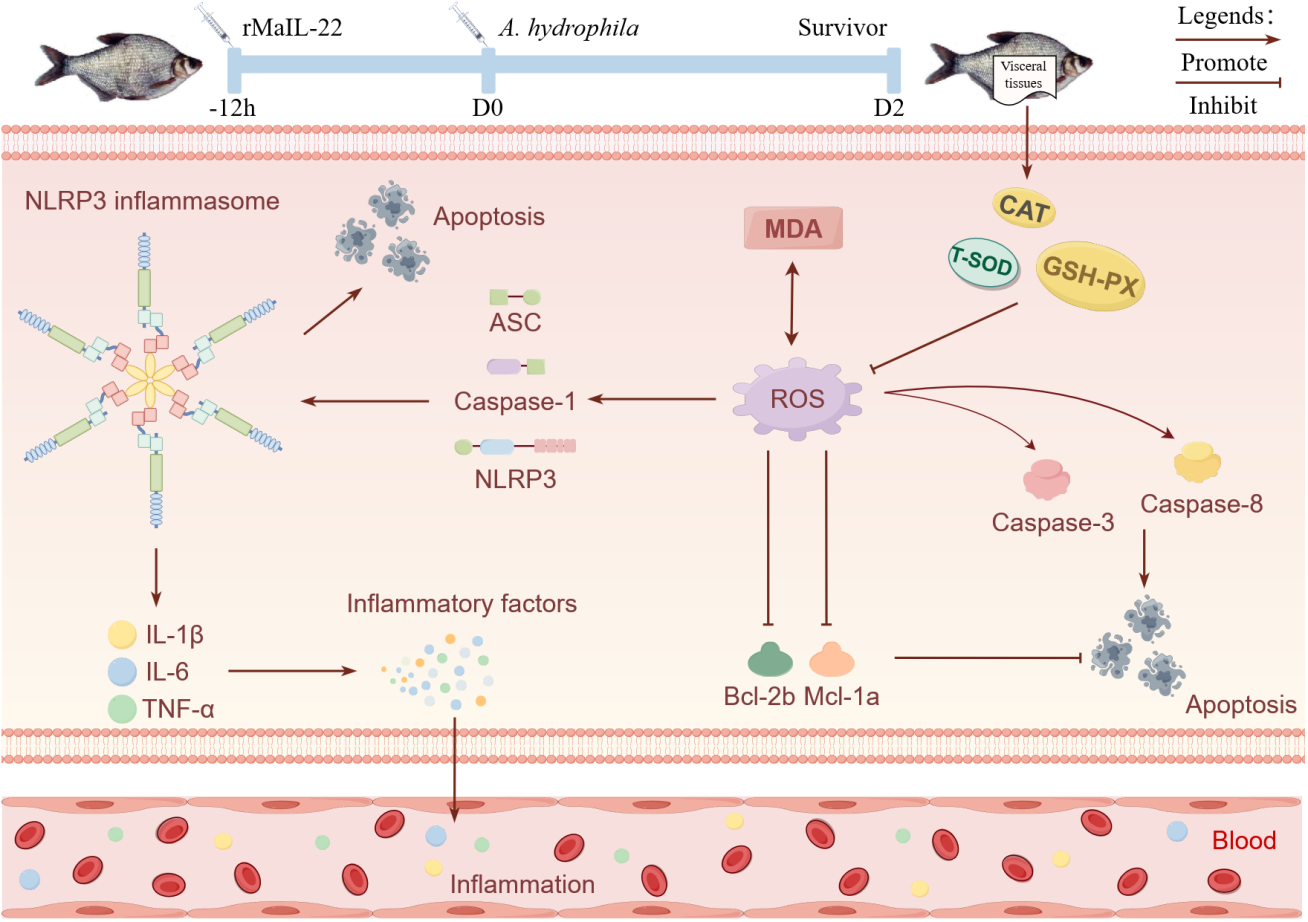
Schematic representation of the enhancement of resistance to *A. hydrophila* in *M. amblycephala* by MaIL-22. Promote and inhibit denote the potential promotion or inhibition of specific biological processes by rMaIL-22. Visceral tissues, blood, inflammation, and apoptosis indicate the primary tissues and physiological processes in which rMaIL-22 may exert its effects. This figure was drawn via Figdraw.

In this study, we identified homologues of IL-22 in *M. amblycephala*, an economically important freshwater species in China, and explored its role in defense against *A. hydrophila*. MaIL-22 exhibited lower sequence identity yet retained conserved structural features in comparison to other teleosts IL-22 homologues. Furthermore, MaIL-22 was implicated in the anti-*A. hydrophila* immune response in *M. amblycephala*. Subsequent experiments demonstrated that rMaIL-22 effectively enhanced *M. amblycephala*’s ability to combat *A. hydrophila* infection. This enhancement was attributed to rMaIL-22’s inhibition of MDA activity and scavenging of ROS, achieved by bolstering antioxidant enzyme activity. Additionally, rMaIL-22 further inhibited the activation of the NLRP3 inflammasome. These actions collectively led to a reduction in the expression of inflammatory factors, the inhibition of apoptosis, and ultimately, the attenuation of tissue injury (**Figure 10**). Overall, this study not only provides new insights for understanding the immune function of IL-22 in fish, but also provides potential molecular targets for the development of new therapeutics and molecular breeding strategies against bacterial diseases.

## Data Availability

The datasets presented in this study can be found in online repositories. The names of the repository/repositories and accession number(s) can be found in the article/**Supplementary Material**.
