# Supplementary material for: Megalobrama amblycephala IL-22 attenuates Aeromonas hydrophila induced inflammation, apoptosis and tissue injury by regulating the ROS/NLRP3 inflammasome axis
Source: Front Immunol. 2024 Aug 15;15:1447431. doi: 10.3389/fimmu.2024.1447431 (PMC11358693; doi:10.3389/fimmu.2024.1447431)
Supplement: Supplementary file 1 [file Table1.doc]

**Table S1** Primers used in this study.

| Primer name | Primer sequence (5′-3′) | Gene ID | Usage |
| --- | --- | --- | --- |
| IL-22-32a-F | CCGGAATTCATGAAGTTCCTAACTCTGCTT | XM_048156469.1 | PCR |
| IL-22-32a-R | CCGCTCGAGTCACATTCTTGCAGAGC |  |  |
| IL-22-F | GGGGGATTACGCCAAAGGTA | XM_048156469.1 | qPCR |
| IL-22-R | TCCTCGCAGACGAGATTTGG |  |  |
| NLRP3-F | TGGAGTTGTGTCTCTCCAACG | XM_048194926.1 | qPCR |
| NLRP3-R | ACAGCACTTGTTGGACAGGTT |  |  |
| ASC-F | TGTGCACAGAAACACCAGTGA | XM_048194983.1 | qPCR |
| ASC-R | CTTTAGTTTGTCTGTGGTCAGATCC |  |  |
| Caspase-1-F | AGATCAAAACCGTGTCGGGG | XM_048195537.1 | qPCR |
| Caspase-1-R | TTCATACCCTGTGCCGTGAG |  |  |
| IL-1β-F | AGCGATTTGCCTCTGTACAAC | XM_048181166.1 | qPCR |
| IL-1β-R | TCCTTCTCCCAGAGCCGTT |  |  |
| TNF-α-F | GAGCCATTTGGTTTTGCGCT | XM_048201192.1 | qPCR |
| TNF-α-R | GATCGTCACTGTCAGACCCG |  |  |
| IL-6-F | TTGTCACACTGGCAGTTTCC | XM_048203704.1 | qPCR |
| IL-6-R | TCCTGGTCGTTCAGAGGACT |  |  |
| Bcl-2b-F | AATATCTGAACGGGCCGCTG | XM_048163874.1 | qPCR |
| Bcl-2b-R | CTTCCACACTCCCGCAGAAT |  |  |
| Mcl-1a-F | TTAAGACCGGGAACGAACGG | XM_048203388.1 | qPCR |
| Mcl-1a-R | GAACCGAGCTCCTTCTCGTC |  |  |
| Caspase-3-F | TAACCGCTTTGTCGTCGGAA | XM_048197464.1 | qPCR |
| Caspase-3-R | TTGTCTCTTGACTCGCACGG |  |  |
| Caspase-8-F | GCCCGCGAAGAGAAAGTAAC | XM_048200589.1 | qPCR |
| Caspase-8-R | GTTGGTGCATGCGTCAGTTC |  |  |
| *18S rRNA*-F | CCGGACACGGAAAGGATTGA | AB860215.1 | qPCR |
| *18S rRNA*-R | GCCGGAGTCTCGTTCGTTAT |  |  |

Notes: IL-22, interleukin 22; NLRP3, the nucleotide-binding and oligomerization domain (NOD)-like receptor pyrin domain-containing; ASC, apoptosis-associated speck-like protein containing a CARD; Caspase-1, cysteinyl aspartate specific proteinase-1; IL-1β, interleukin 1β; TNF-α, tumor necrosis factor α; IL-6, interleukin 6; Bcl-2b, B-cell lymphoma-2b; Mcl-1a, myeloid cell leukemia 1a; Caspase-3, cysteinyl aspartate specific proteinase-3; Caspase-8, cysteinyl aspartate specific proteinase-8. *18S rRNA*, 18S ribosomal RNA.
